# Supplementary material for: Global hotspots and correlates of emerging zoonotic diseases
Source: Nat Commun. 2017 Oct 24;8:1124. doi: 10.1038/s41467-017-00923-8 (PMC5654761; doi:10.1038/s41467-017-00923-8)
Supplement: Supplementary file 1 — Supplementary Information [file 41467_2017_923_MOESM1_ESM.pdf]

Supplementary Methods

Introduction

PubCrawler is a high-resolution spatial dataset representing estimated "reporting effort" that serves as a background weight for our model.

"Reporting bias" refers to bias in a model that is due to the unequal distribution of reporting effort, leading to uneven detection probability for the outcome of interest (in our case EID events). We followed previous authors and reasoned that variation in detection probability of disease events would be primarily determined by "search effort"; namely, the extent to which disease events have been looked for.[1,2] Since search effort is difficult to estimate directly for infectious diseases at a global scale, we further reasoned that search effort would be a function of "reporting effort", which is the extent to which disease events are "reported" to become part of the scientific literature. This is appropriate here since our observations of disease events are similarly drawn from the scientific literature.

Previous studies have similarly recognized this issue when modeling infectious disease occurrence, and various methods have been used to estimate the distribution of reporting effort. In certain cases, presence-only data may be supplemented with data on known sampling bias, or data from other planned sampling efforts.[3] A previous study using an earlier version of the dataset used in the present study used locations drawn from the Journal of Infectious Disease as a measure of reporting effort.[4] An index was created using the country of residence of every author from all JID articles since 1973. This country-level measure of reporting effort was then downscaled to match the spatial grid used in the analyses (~100 x 100km) and included as a covariate in a logistic regression model. Similarly, in their study of water-associated infectious disease events, Yang et al., (2012) searched PubMed for "infectious disease" and "[country name]" and recorded the yearly counts at the country level for 1991 through 2008 [5]. In their spatial Poisson model, these country level publication count estimates were again downscaled to match the spatial grid used in their analyses and included as an offset.[5]

In our analyses, we sought to improve on these methods by creating a better-than country level reporting effort dataset. Country-level measures of reporting effort may be limited as a correction for more fine-grain bias in the detection of disease events in people. Furthermore, in the case of Jones et al., (2008), the country of residence of authors may not be a good proxy for the locations in which studies have actually occurred. To solve these issues, we adapted methods from ecology that are now routinely used when modeling species distributions from "presence-only" data.

In "presence-absence" models, where both positive (i.e., presence/occurrence records) and negative (i.e., absence) outcome samples are gathered from the same dataset (e.g. systematized surveys for species), both presence and absence records are drawn from the same covariate space and can be compared directly. However, the same is not true for datasets involving only observations of an event (i.e., presence-only). In such circumstances, presence records are typically contrasted with randomly generated records, often termed "background data" or (somewhat misleadingly) "pseudo-absence" records. However, while it is

reasonable to compare a distribution of observations against a random distribution, the set of "background data" provided to the model may innately differ from the covariate space of search effort, and this can cause poor model performance and/or spurious associations.[1-3] Weighting or stratifying background points by a proxy or estimate of detection probability can ameliorate this problem. By balancing the distribution of covariates in the background sample and a hypothetical null outcome sample, the divergence between those measured by the model should be due to the effect of interest.[1-31]

Materials and Methods

All code used to generate the dataset is available on GitHub [6].

To estimate reporting effort and integrate it with our models, we wrote a series of scripts in Python and R to identify place names in the PubMed Central Open Access Subset (PMCOAS), and aggregate them to the spatial grid used in our analyses. This section describes the current capabilities of the *PubCrawler* Python package, and then details the workflow used in the creation of the reporting effort layer for this study.

We developed three components for our workflow:

- *PubCrawler*, a Python package, which provides functionality for extracting information from PMCOAS.
- *Annie*, a generalized text annotation Python package, first developed for the GRITS (Global Rapid Identification of Threats) natural language processing tool.[7] *PubCrawler* uses *Annie*'s GeonameAnnotator() class, and a modified method from that class, to identify toponyms in article text.
- *pubcrawler2hotspots*, a set of R scripts bundled as a package, which aggregates extracted toponyms to the spatial grid used in our main model, and fits a boosted regression tree model to the output.

Managing Articles

PubCrawler, written in Python 3.4, consists of a number of classes and scripts that facilitate the extraction of data from PMCOAS. *PubCrawler* stores data, including the PMCOAS and GeoNames toponyms (described below) in Mongo databases. Scripts are included to initialize these databases in the required format and ingest the raw data.

Articles in the PMCOAS are available in NXML (National Libraries of Medicine XML) format, a schema that defines a number of entities, including article identifiers, publication type, indicators for keywords, and publication dates. These are stored in the database with the contents of the file as a property named "nxml" and the name of the file as Mongo's "_id" property.

PubCrawler defines the class Article(), which takes an article from the Mongo database. This class has methods defined for accessing the article's publication IDs, publication dates, article type (e.g. "research-article"), title, and keywords (if any), as well as the text from any named tags in the XML document.

A separate module defines three “extractor” functions, described below, along with a number of auxiliary functions, to manage writing the extracted information to the documents in the database. Some of the extractor functions use *Annie* internally.[7]

The script “crawler.py” provides the core functionality of the *PubCrawler* package, iterating across batches of articles in parallel, extracting and saving metadata using specified extractor functions (described below).

After toponyms and other information are extracted, the script “export.py” iterates through articles (optionally specifying a subset via a Mongo query), writing a CSV file per article in a directory (in subdirectories of 10,000) with all extracted locations, plus pertinent metadata.

Extracting Metadata

The PMCOAS contains everything published in participating journals, including research articles, reviews, errata, figures, and commentary.[8] For our analyses, we only exported data for research articles. The extract_meta() function records this information from NXML tags and stores it in the database. The script “create_index.py” tailors this information to facilitate processing the large volumes of data.

Extracting Infectious Disease Terms

The PMCOAS includes articles on “biomedical and life sciences”, broadly defined.[8] We restricted our search to articles related to infectious diseases. To do this, we first tried using keywords as they are represented in NXML documents; however, the <kwd> element is not consistently applied throughout the OAS. In a random sample of 10,000 articles, 50.3% (95% CI [49.4%, 51.3%]) contained the <kwd> element, and these keywords may come from various ontologies (such as MeSH) or be author-created.[9] For each article, We searched the full body text for diseases caused by infectious agents in the Human Disease Ontology (1392 keywords).[10] Accordingly, the function extract_disease_ontology_keywords() uses the Human Disease Ontology (in OWL format) to return a list of keywords found in an article, which are recorded in the database. The infectious disease terminology extractor uses *Annie*’s KeywordAnnotator() class.

Extracting Toponyms

PubCrawler’s toponym resolution uses *Annie*’s GeonameAnnotator() class in conjunction with some amended methods from that class. The algorithm uses the GeoNames database as its gazetteer, which consists of over 10,000,000 named locations, including 2,800,000 populated places.[11] GeoNames metadata includes location, alternate names in multiple languages, geographic “feature class”. Toponyms are resolved in a multi-stage process:

- Candidate locations are extracted by searching 1- through 7-word n-grams (contiguous sequences of words constructed with a “moving window” through the text, internally termed “spans”) for text matches in the GeoNames database, using both the “name” and “alternatenames” fields (GeoNames’s “alternatenames” generally includes the toponym in a variety of languages, and with different permutations accent markings and other textual features). Where a span matches multiple GeoName entities, the list of “alternateLocations” is recorded. In addition, alternate locations that match different

spans (e.g. “Harare Province” has “Harare” as an alternate name; these would be listed, as would the city “Harare”).

- For each location, “features” are extracted. Each feature is a property of the location or of its place in the text, and is assigned a score between 1 and 100. Features, scores, and weights (below) were developed through expert evaluation and empirical observation of results from a small test article set. Features used in the current version are as follows (specific scoring criteria can be found [in the code]):
    - population_score – categories based on population size
    - synonymity – categories based on the number of alternate names for a feature
    - num_spans_score – categories based on the number of times the location occurs in the text
    - short_span_score – penalties applied for matches under 4 and 5 characters
    - NEs_contained – a score based on whether the spans matching the text are flagged as named entities by Annie’s named entity recognizer
    - distinctness – a score based on the number of alternate locations matched for a word
    - max_span_score – a score assigned based on the length of text matching the location, assuming that longer text allows for greater specificity
    - feature_code_score – a score given to features of certain classes, including continents
    - canonical_name_used – a score given if any of the text matches for a location use its primary specified name
- Feature scores are weighted post-assignment and used in an algorithm to filter false positives and disambiguate ambiguous toponyms. First, toponyms with a score lower than 50 are removed. The remaining locations are passed to an algorithm that only returns the combination of toponyms for non-overlapping text spans which maximizes the summed feature weights for the document.
- The complete and culled sets of toponyms for the article are stored, referencing the GeoNames database.

Spatial Aggregation Workflow

The R package *pubcrawler2hotspots* contains the code for the spatial aggregation and model fitting workflows described below, used to generate the reporting effort layer in this study.

PubCrawler was run on the PMCOAS articles. Locations were exported to CSV for all research articles whose body text matching Human Disease Ontology keywords. Before aggregation, additional filtering was conducted on locations.

- Continents were excluded. Continents are frequently matched, but because each GeoName is assigned a single set of coordinates in the centroid of the continent, their inclusion creates a few spurious high-value outliers. Countries are excluded for the same reason. Lower-level administrative divisions did not cause as severe a problem due to the coarse target resolution, and so were not excluded.
- A short list was compiled of 20 frequently-matched, obviously-spurious terms which appeared despite the culling process, here listed in descending order of frequency: "American River", "Candida", "Research", "Centre", "Sigma", "Normal", "Middle", "Tukey", "The World", "Golgi", "Male", "Horizontal", "Teaching Hospital", "Cancer", "Altogether", "Delta", "Excel", "Chicken", "Basic", and "Scheme".

- Locations in the GeoNames classes “spot, building, farm” and “undersea” were excluded, as were zero-population locations.

Each article was assigned a weight of 1, which was distributed uniformly across the remaining locations. These weights were aggregated to the grid used for the study's main model.

Model Fitting

The raw layer of weighted counts was not appropriate for use directly in weighting a model. Our data source constitutes only a sample of the research literature, and errors of omission and commission in our toponym resolution add noise to that sample. Practically speaking, this led to a number of zero-count grid cells in low-publication areas and outlying high-count grid cells and resulting in overdispersion (the layer’s variance, 4919.2, is much higher than the mean of 7.5). Fitting a model allows us to fill in these gaps and smooth this distribution. We fit a Poisson boosted regression tree to the aggregated layer, and used the predicted values for grid cells as our reporting effort layer.

Because the data exhibited overdispersion and excess zeros, we explored GLM frameworks for zero-inflated and overdispersed data during our fitting process:

- Poisson GLM, which found all variables be significantly associated with the outcome, but was inappropriate for use because of overdispersion;
- Quasipoisson GLMs, which had large dispersion parameters and non-significant p-values;
- glm.nb() function from the pscl package, for a negative binary GLM, which failed to fit;
- Zero-inflated Poisson models using the zeroinfl() function from the boot package, which encountered problems fitting.
- In case the large number of zeroes was caused by a lack of GeoNames entities in the zero grid cells, we created a variable by summing the number of eligible GeoNames entities in all grid cells (using the same categories as were used to match locations in PubMed Central text). Including this as a coefficient and as an offset in the models did not solve the problems the GLM modules encountered.

We posited that the fit problems are due to the high number of zeroes (approximately 78% of grid cells were zero) and high-valued outliers among grid cells, which do not conform to the distributional requirements the GLMs and their fitting algorithms. Because of this, we discarded the GLMs.

We selected BRTs largely because they are able to return fairly accurate predictions — as long as they are predicting values which occur within the covariate space in which they were trained — and are robust to data which would be problematic or assumption-violating for other modeling methods, including sparse data and non-normally distributed data.

Although the predominant R packages for boosted regression trees do not include variants of zero-inflated Poisson or negative binomial regression, BRTs flexibility permits them to capture the higher variance that results from overdispersion/excess zeroes. Since BRTs aggregate smaller models, fit to subsections of covariate space, values for covariates (and thus λ) may vary between different subsections of covariate space. (The prediction layer from the Poisson BRT indeed exhibits similar overdispersion to the raw layer, with a mean of 7.9 and variance of 3232.2.)

Predictor Datasets

We selected predictor datasets based on a priori hypotheses about mechanisms shaping research effort.

The following predictors were read in at grid-cell resolution:

- Human population size, using the GRUMP dataset also included in the main model. We seek to model research on human infectious disease, and such research is also conducted *by* humans.
- Average travel time to cities with populations > 50,000. Research may be conducted more often in more accessible areas.
- Percentage of grid cell of urbanized land, from the EarthEnv dataset included in the main model. Urbanization may serve as a broad proxy for factors increasing the likelihood of research being conducted, including the presence of funding and resources, above and beyond sheer population density.

The following predictors were available at country level. We associated them with JSON shapefiles and projected them to the study grid, weighting *per capita* variables by population per grid cell.

- Per capita DALY rates from all causes (WHO). More research may be conducted where there is thought to be a higher burden of disease.
- Per capita health expenditure (WHO). Greater health care expenditure in an area may lead to more research being conducted in that area.
- Per capita GDP (UN). Places with more economic activity may conduct more research. Lower-GDP countries generally have a higher burden of infectious disease, and so may see more research conducted in the aggregate, but DALY rates are included in the model.

The Poisson BRT module used requires that the outcome vector to be an integer, but our counts were weighted, so we rounded the aggregated output values per grid cell to whole numbers. The counts were large enough that this made no meaningful difference to the distribution (R^2 = 1).

The boosted regression tree was fit using the “gbm.step()” function in the R package “dismo”. The tree complexity used in the final model was 3; the learning rate was 0.01; the bag fraction was 0.75; the number of trees added per iteration was 50.

Results

Of the 1,266,085 articles in the PMCOAS, 931,087 (73.5%) were research articles, and 204,097 matched our infectious disease keyword search and (16.1%) matched our infectious disease search query. 157,779 articles matched both criteria, and contained matched locations. The mean total deviance for the publication model was 56.5, and the residual deviance was 15.2; the percentage explained was 73%.

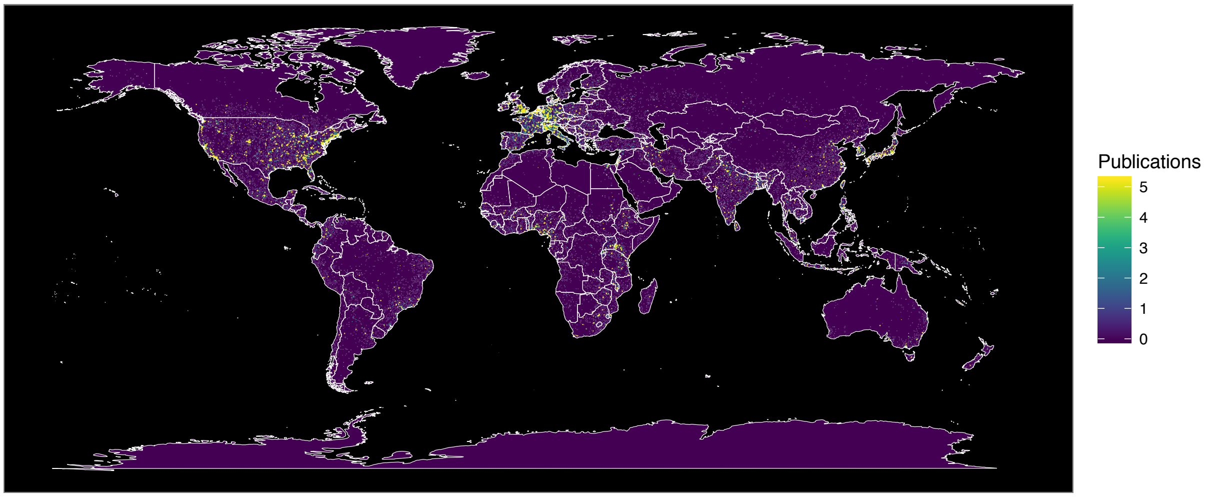

Supplementary Figure 1. Locations extracted from publications matching infectious disease keywords. Toponyms with non-zero populations were weighted uniformly, normalized by publication, and aggregated to a 0.25° grid. Before plotting, toponym count was truncated at the 90th percentile, to prevent outlier grid cells from skewing color palette scaling.

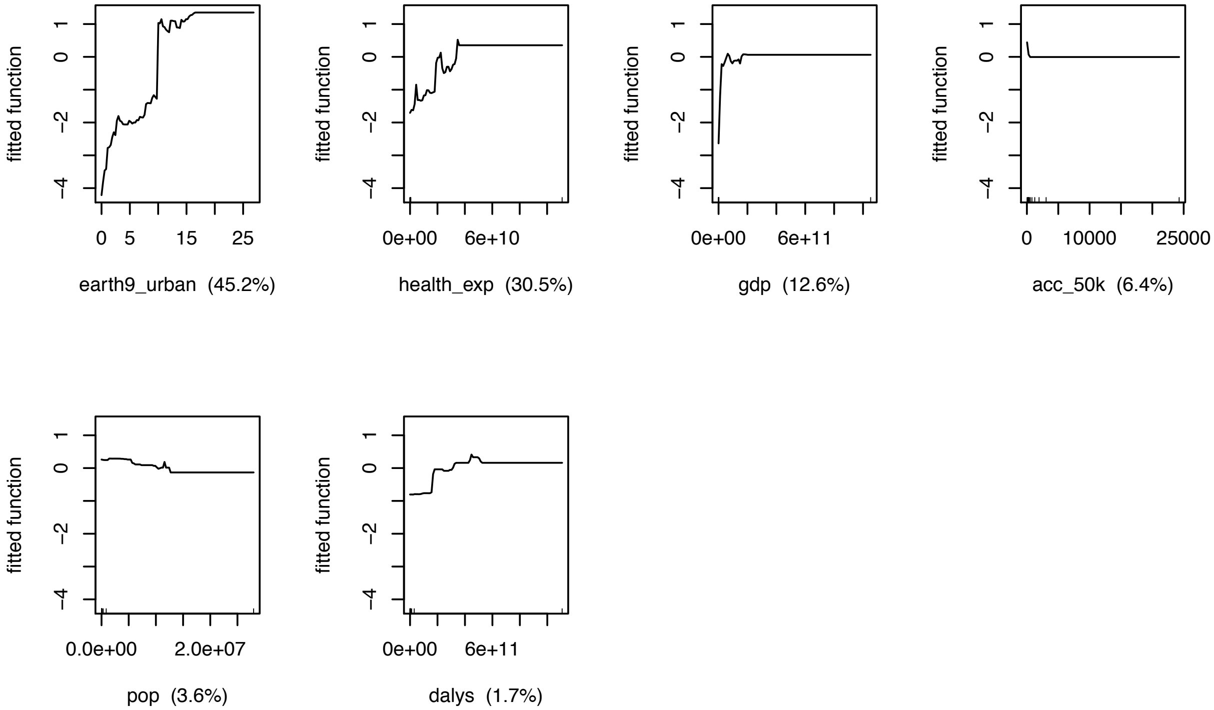

Supplementary Figure 2. Partial dependence plot for the BRT model used to smooth the publication layer, plus relative influence of variables.

Our reporting effort layer is statistically significantly associated with the layer used by Jones et al.[4] when aggregated to country level (McFadden's Pseudo R^2 = 0.78 and $p < 0.005$ computed with a univariate Poisson glm). Since the new report effort layer represents a new process that

is theoretically aligned with our outcome of interest at a smaller-than-country resolution, and produces a similar distribution at country level as the previous method in [4], we think it is a meaningful advancement in disaggregating infectious disease reporting effort past country level. Future research should focus on improving the natural language processing algorithms used to create the measure, and create gold standard datasets to test the accuracy at publication level.

Supplementary Note 1

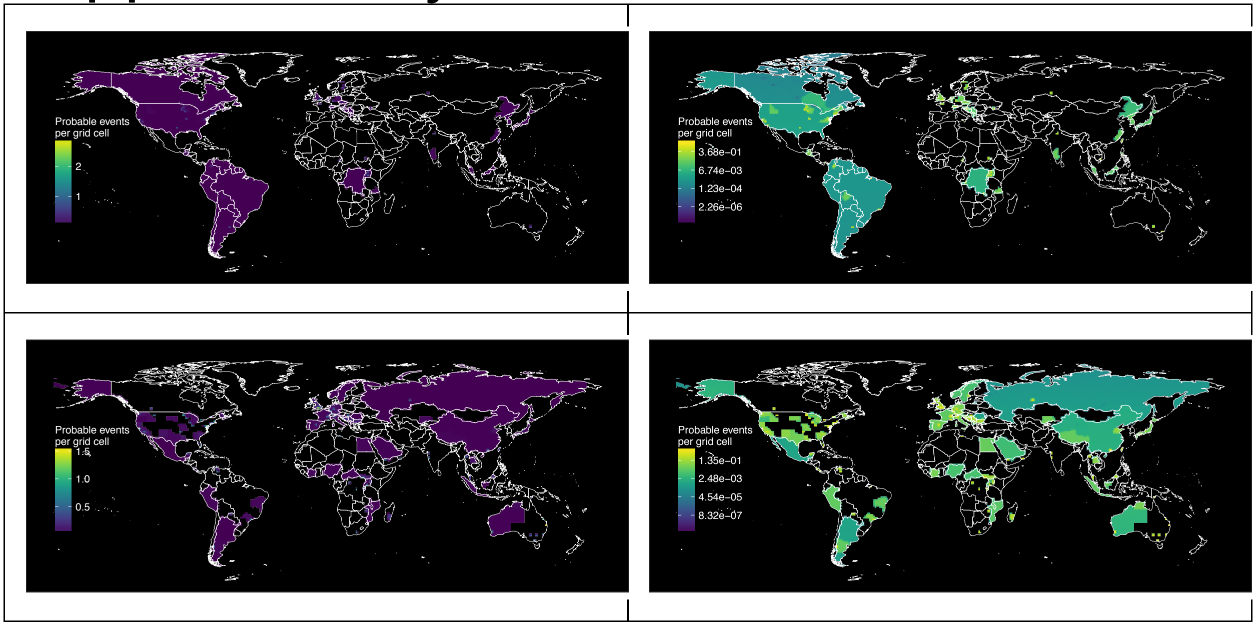

Supplementary Figure 3. Shapefiles for wildlife-origin zoonotic EID events, summed to the study grid. The top row shows pre-1970 events, excluded from analyses; the bottom row shows post-1970 events included in analyses, as used for the "unweighted" model's presence samples. The left column scales colors linearly across values, whereas the right column scales the color palette across log-transformed values to better reveal large events. Each event is distributed across the grid cells its known occurrence area encompasses such that the value represents the probability that it occurred in that cell (here assumed to be across land area, as in the "unweighted" model). An event overlapping only one grid cell would contribute a value of 1 to that grid cell; an event covering the entirety of Russia would contribute a small fraction to each grid cell. The summed value is used to weight bootstrap presence samples. (See Methods for more details on sampling regime.)

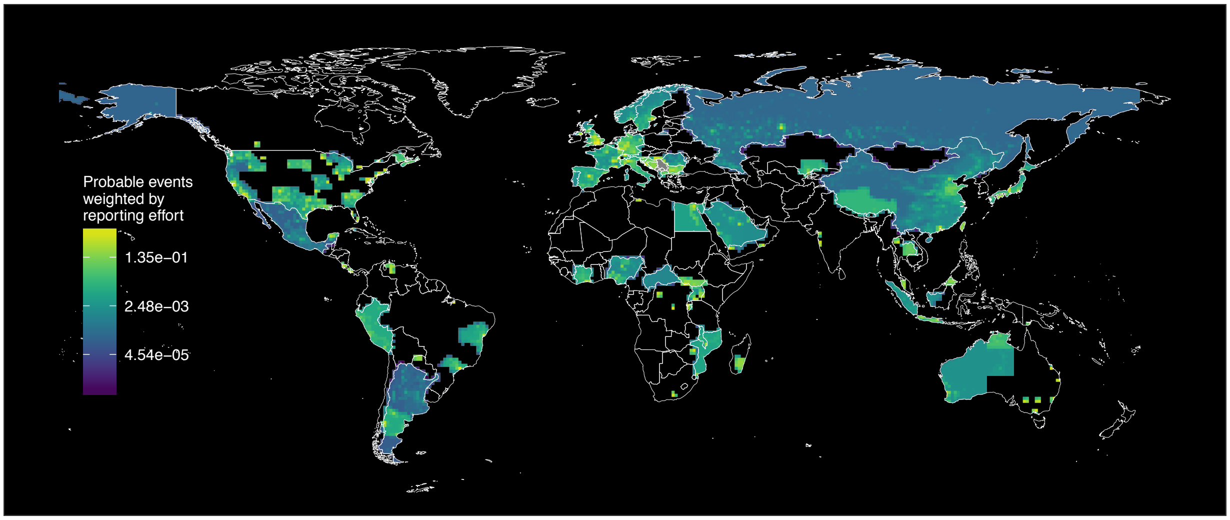

Supplementary Figure 4. EID events post-1970, summed to the study grid in the manner described above but weighted by reporting effort, instead of land area, as in the "weighted" model. The color palette is scaled logarithmically to better show differences on the low end of the distribution. These represent the presence sample

for the weighted model (compare to bottom-right quadrant of Supp. Figure 3.1 for unweighted model's events with the same color palette scaling).

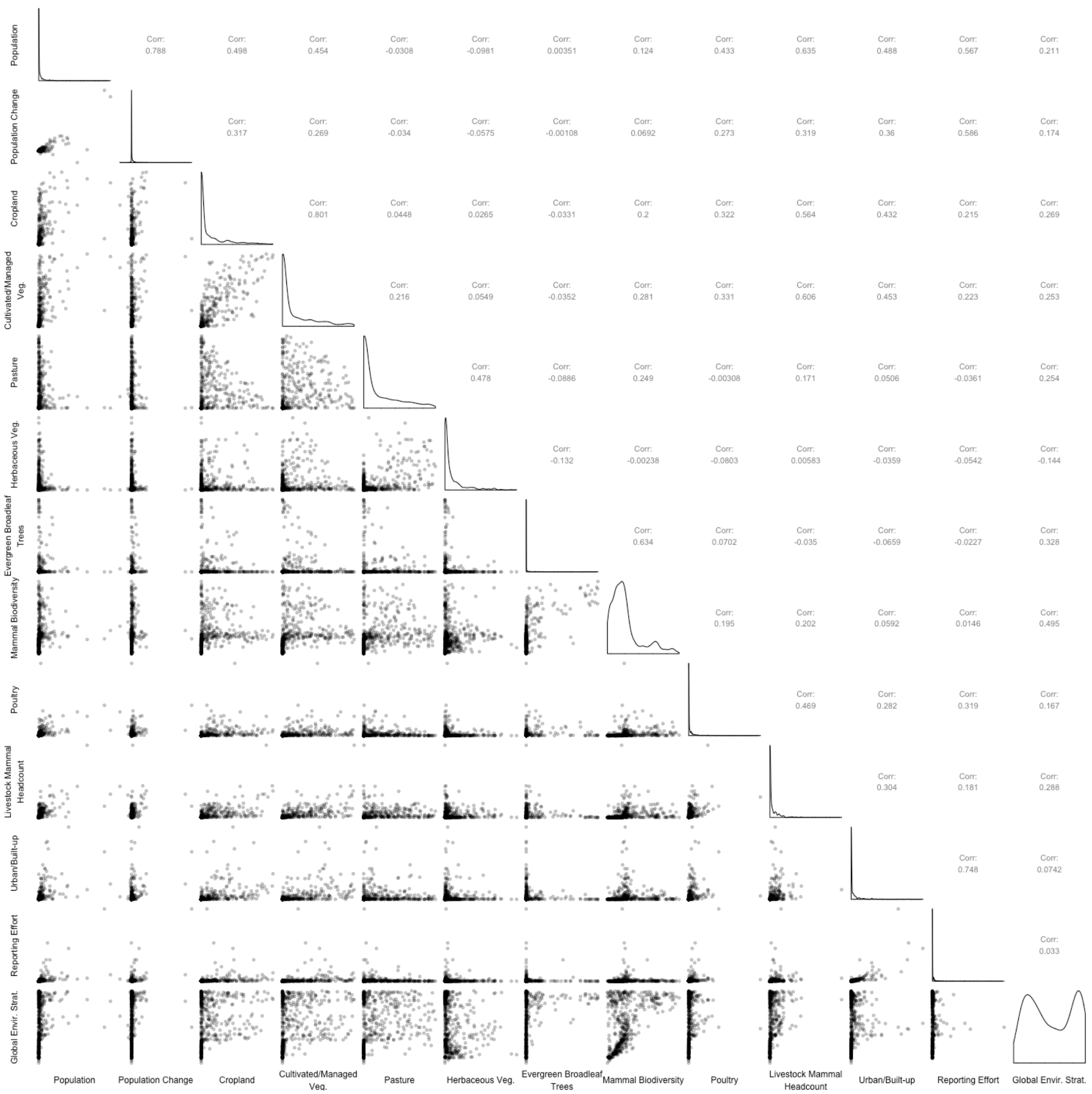

Supplementary Figure 5. Scatterplot matrix and correlation coefficients for variables in the model which exhibit a correlation coefficient of ≥ 0.5 with any other variable, and additional variables of interest.

Supplementary Note 2

Additional Output from Weighted Model

Variable 1	Variable 2	Interaction Strength Quantile		
		**0.25**	**0.5**	**0.75**
Pasture Change	Cropland Change	0.15	0.31	0.81
Global Envir. Strat.	Pasture Change	0.12	0.28	0.6925
Livestock Mammal Headcount	Mixed/Other Trees	0.11	0.28	0.39
Cropland Change	Cropland	0.12	0.27	0.7
Shrubs	Mixed/Other Trees	0.1125	0.265	0.3275
Pasture Change	Cropland	0.12	0.26	0.585
Mixed/Other Trees	Cropland Change	0.11	0.25	0.57
Mammal Biodiversity	Pasture Change	0.1	0.25	0.67
Urban/Built-up	Pasture Change	0.1	0.245	0.555
Mixed/Other Trees	Pasture Change	0.1225	0.24	0.4975

Supplementary Table 1. Quantiles for BRT interaction strength, as assessed by the gbm.interactions() function from the dismo *package, across the 1000 runs of the weighted model.*

The following figures show the relative influence and partial dependence plots, and top ten interactions, from the unweighted model.

Supplementary Note 3

Output from Unweighted Model

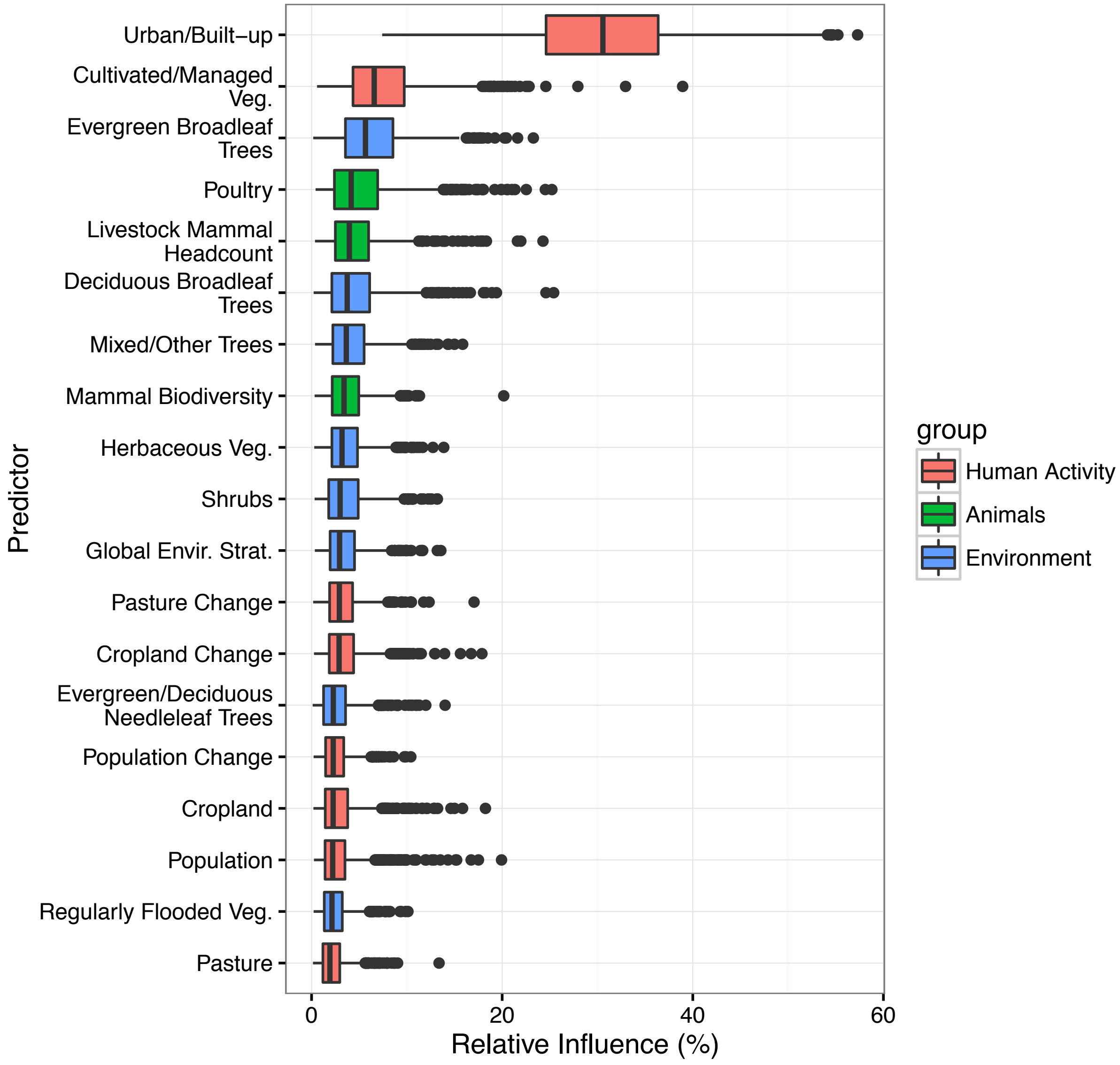

Supplementary Figure 6. Relative influence of predictors in unweighted model.

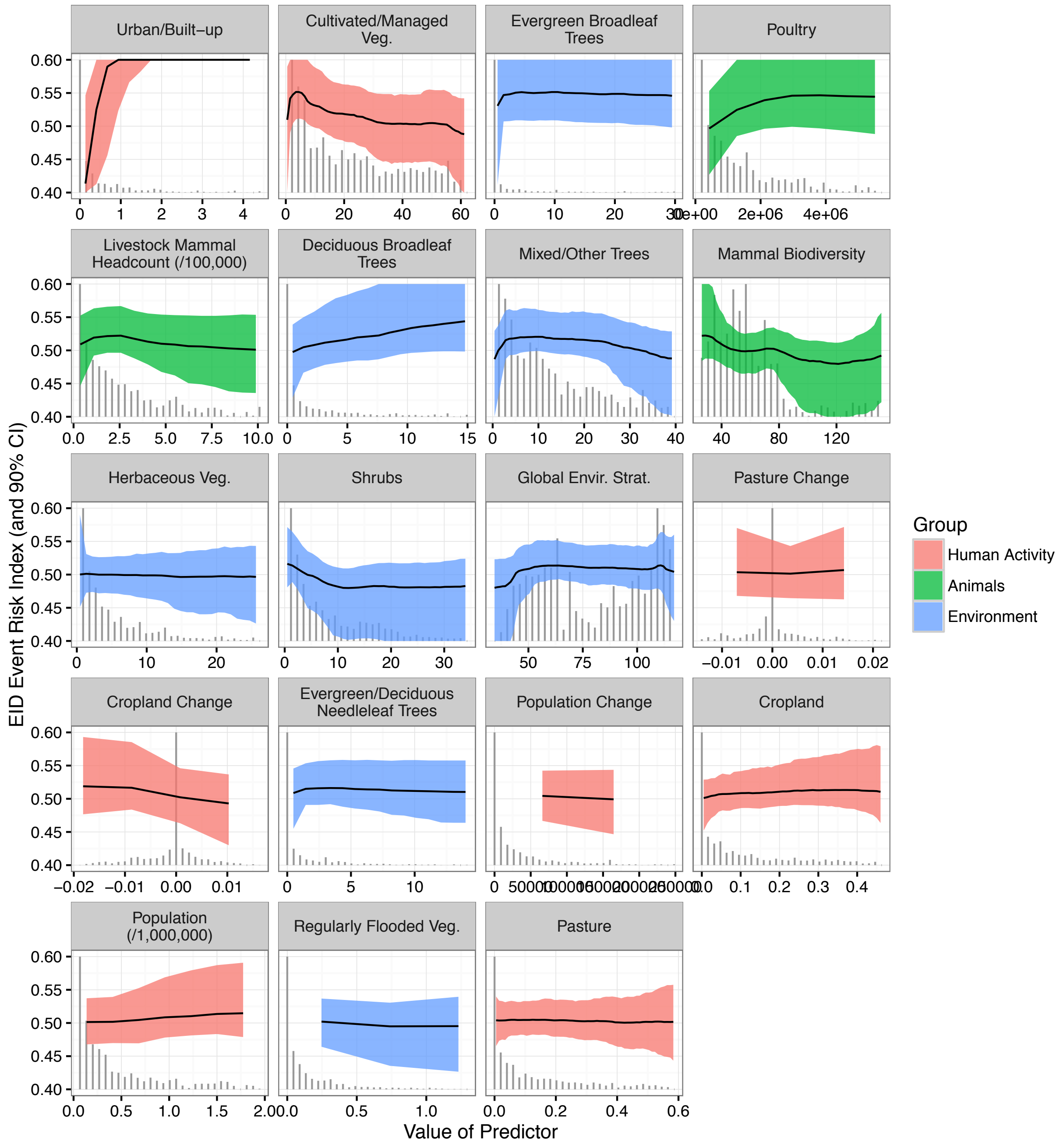

Supplementary Figure 7. Partial dependence plots for unweighted model.

Variable 1	Variable 2	Interaction Strength Quantiles		
		**0.25**	**0.5**	**0.75**
Regularly Flooded Veg.	Herbaceous Veg.	0.41	0.44	0.51
Urban/Built-up	Pasture Change	0.2	0.42	1.01
Cultivated/Managed Veg.	Cropland Change	0.19	0.41	0.885
Urban/Built-up	Deciduous Broadleaf Trees	0.16	0.39	0.94
Regularly Flooded Veg.	Cropland	0.27	0.38	0.4
Urban/Built-up	Cultivated/Managed Veg.	0.17	0.37	0.84
Pasture Change	Cropland Change	0.18	0.36	0.6975
Shrubs	Pasture Change	0.1525	0.35	0.585
Herbaceous Veg.	Pasture Change	0.185	0.35	0.77
Mammal Biodiversity	Cultivated/Managed Veg.	0.15	0.35	0.7

Supplementary Table 2. Quantiles for BRT interaction strength, as assessed by the gbm.interactions() function from the dismo *package, across the 1000 runs of the weighted model.*

Supplementary References

1. Phillips, S. J. *et al.* Sample selection bias and presence-only distribution models: implications for background and pseudo-absence data. *Ecological Applications* **19,** 181–197 (2009).
2. Massin, M. B., Jiguet, F. & Albert, C. H. Selecting pseudo-absences for species distribution models: how, where and how many? *Methods in Ecology …* (2012). doi:10.1111/j.2041-210X.2011.00172.x
3. Dorazio, R. M. Accounting for imperfect detection and survey bias in statistical analysis of presence-only data. *Global Ecology and Biogeography* **23,** 1472–1484 (2014).
4. Jones, K. E. *et al.* Global trends in emerging infectious diseases. *Nature* **451,** 990–993 (2008).
5. Yang, K. *et al.* Global Distribution of Outbreaks of Water-Associated Infectious Diseases. *PLOS Negl Trop Dis* **6,** e1483 (2012).
6. Allen, T. & Breit, N. ecohealthalliance/pubcrawler: 'Global correlates' paper. (2016). doi:10.5281/zenodo.163676
7. Huff, A. G., Breit, N., Allen, T., Whiting, K. & Kiley, C. Evaluation and Verification of the Global Rapid Identification of Threats System for Infectious Diseases in Textual Data Sources. *Interdiscip Perspect Infect Dis* **2016,** 5080746 (2016).
8. U.S. National Library of Medicine. PMC FAQs. *PubMed Central* (2015). Available at: https://www.ncbi.nlm.nih.gov/pmc/about/faq/. (Accessed: 14 October 2016)
9. U.S. National Library of Medicine. *Journal Publishing Tag Library NISO JATS*. (U.S. National Library of Medicine, 2014).
10. Kibbe, W. A. *et al.* Disease Ontology 2015 update: an expanded and updated database of human diseases for linking biomedical knowledge through disease data. *Nucleic Acids Res.* **43,** D1071–8 (2015).
11. Wick, M. & Boutreux, C. GeoNames.
