# Supplementary material for: Global hotspots and correlates of emerging zoonotic diseases
Source: Nat Commun. 2017 Oct 24;8:1124. doi: 10.1038/s41467-017-00923-8 (PMC5654761; doi:10.1038/s41467-017-00923-8)
Supplement: Supplementary file 3 — Description of Additional Supplementary Files [file 41467_2017_923_MOESM3_ESM.pdf]

Description of Supplementary Files

File name: Supplementary Data 1
Description: Table of EID database used in statistical model.
